# Designing financial-incentive programmes for return of medical service in underserved areas: seven management functions

**DOI:** 10.1186/1478-4491-7-52

**Published:** 2009-06-26

**Authors:** Till Bärnighausen, David E Bloom

**Affiliations:** 1 Africa Centre for Health and Population Studies, University of KwaZulu-Natal, Mtubatuba, South Africa; 2 Harvard School of Public Health, Harvard University, Boston, Massachusetts, USA

## Abstract

In many countries worldwide, health worker shortages are one of the main constraints in achieving population health goals. Financial-incentive programmes for return of service, whereby participants receive payments in return for a commitment to practise for a period of time in a medically underserved area, can alleviate local and regional health worker shortages through a number of mechanisms. First, they can redirect the flow of those health workers who would have been educated without financial incentives from well-served to underserved areas. Second, they can add health workers to the pool of workers who would have been educated without financial incentives and place them in underserved areas. Third, financial-incentive programmes may improve the retention in underserved areas of those health workers who participate in a programme, but who would have worked in an underserved area without any financial incentives. Fourth, the programmes may increase the retention of all health workers in underserved areas by reducing the strength of some of the reasons why health workers leave such areas, including social isolation, lack of contact with colleagues, lack of support from medical specialists and heavy workload.

We draw on studies of financial-incentive programmes and other initiatives with similar objectives to discuss seven management functions that are essential for the long-term success of financial-incentive programmes: financing (programmes may benefit from innovative donor financing schemes, such as endowment funds, international financing facilities or compensation payments); promotion (programmes should use tested communication channels in order to reach secondary school graduates and health workers); selection (programmes may use selection criteria to ensure programme success and to achieve supplementary policy goals); placement (programmes should match participants to areas in order to maximize participant satisfaction and retention); support (programmes should prepare participants for the time in an underserved area, stay in close contact with participants throughout the different phases of enrolment and help participants by assigning them mentors, establishing peer support systems or financing education courses relevant to work in underserved areas); enforcement (programmes may use community-based monitoring or outsource enforcement to existing institutions); and evaluation (in order to broaden the evidence on the effectiveness of financial incentives in increasing the health workforce in underserved areas, programmes in developing countries should evaluate their performance; in order to improve the strength of the evidence on the effectiveness of financial incentives, controlled experiments should be conducted where feasible).

In comparison to other interventions to increase the supply of health workers to medically underserved areas, financial-incentive programmes have advantages – unlike initiatives using non-financial incentives, they establish legally enforceable commitments to work in underserved areas and, unlike compulsory service policies, they will not be opposed by health workers – as well as disadvantages – unlike initiatives using non-financial incentives, they may not improve the working and living conditions in underserved areas (which are important determinants of health workers' long-term retention) and, unlike compulsory service policies, they cannot guarantee that they will supply health workers to underserved areas who would not have worked in such areas without financial incentives. Financial incentives, non-financial incentives, and compulsory service are not mutually exclusive and may positively affect each other's performance.

## Background

In many countries, one of the main constraints in achieving population health goals is the lack of health workers. The 2004 Joint Learning Initiative (JLI) for Human Resources for Health estimated that "[s]ub-Saharan countries must nearly triple their current numbers of workers by adding the equivalent of one million workers through retention, recruitment, and training if they are to come close to approaching the MDGs [Millennium Development Goals] for health" [1], and the 2006 World Health Report concluded that "[t]he severity of the health workforce crisis in some of the world's poorest countries is illustrated by WHO estimates that 57 of them (36 of which are in Africa) have a deficit of 2.4 million doctors, nurses and midwives" [2].

Interventions to alleviate health worker shortages in medically underserved areas include selective recruitment of those individuals into health care education who are most likely to work in such areas, training specifically for underserved practice, improvements in working or living conditions in underserved areas, compulsion or incentives [3]. The topic of the present article is financial incentives for return of medical service in underserved areas: a health worker enters into a contract to practise for a number of years in an underserved area in exchange for a financial payoff.

Table 1 shows the characteristics of five different types of financial-incentive programmes that have been described in the literature [4-6]: service-requiring scholarships ("conditional scholarships") (e.g. [7-9]), educational loans with service requirement (e.g. [10]), service-option educational loans (e.g. [11]), loan repayment programmes (e.g. [12]), and direct financial incentives (e.g. [13]). These programme types differ according to the time a (future) health worker commits to participation (before, during or after completion of health care education), the time when participants receive monetary payments (during or after completion of health care education), spending restrictions on the received payments (for educational purposes only or for any purpose), and the type of obligation (service and/or financial repayment).

**Table 1:** Types of financial-incentive for return of service programmes

**Type of programme**	**Time of commitment**	**Time of money receipt**	**Spending restrictions**	**Type of obligation**
Service-requiring scholarships ("conditional scholarships")	Before the start of health care education or early in the course of health care education	During health care education	Money earmarked for health care education	Service*

Educational loans with service requirement	Before the start of health care education or early in the course of health care education	During health care education	Money earmarked for health care education	Service and financial repayment*

Service-option educational loans	Before the start of health care education or early in the course of health care education	During health care education	Money earmarked for health care education	Service or financial repayment

Loan repayment programmes	After completion of health care education	After completion of health care education, during committed service	Money earmarked to pay back educational debt	Service*

Direct financial incentives	After completion of health care education	After completion of health care education, during committed service	Money can be used for any purpose	Service*

*Programme may have a buy-out option.

All service-option educational loan programmes offer a choice between service and repayment of the financial incentive. The other four types of programmes commonly offer a "buy-out" option. Service-requiring scholarships with a buy-out option are similar to service-option education loans. However, while programme managers of service-option loans normally consider repayment and service equally desirable outcomes, managers of service-requiring scholarships prefer service over buy-out. Buy-out is thus usually more expensive than financial repayment.

All five types of financial-incentive programmes can potentially serve to increase the numbers of health workers in underserved areas through four mechanisms. First, they may increase the supply of those health workers who would have been educated without financial incentive in underserved areas by decreasing the supply in well-served areas. For instance, they may decrease the net emigration flows of nurses and physicians from the developing world to developed countries [14-16]. This first mechanism can take hold if there are health workers who normally would not work in underserved areas, but who are willing to do so in return for a financial incentive.

Second, they may add health workers to the pool of workers who would have been educated without financial incentives and place them in underserved areas. This second mechanism can take hold if there are qualified candidates who normally would not have the means to finance a health care education, but who can afford to do so if they receive financial incentives, and if a country's health care education system can absorb additional students.

Third, financial-incentive programmes may improve the retention in underserved areas of those health workers who participate in a programme, but who would have worked in an underserved area without financial incentive. Retention may increase, for instance, if programme participants fulfil their contracts and contractual obligations are longer than the times they would have remained in an underserved area without financial incentive.

Fourth, programmes may increase the retention of all health workers in underserved areas by improving the supply of health workers to underserved areas and thus reducing the strength of some of the reasons why health workers leave such areas, e.g. social isolation [17], lack of contact with colleagues [18], lack of support from medical specialists [19], or heavy workload [17,18,20]).

We have previously shown that a specific type of financial-incentive programme, scholarships in return for a commitment to deliver antiretroviral treatment in sub-Saharan Africa, is highly cost-beneficial under a wide range of assumptions [21]. In a recent systematic review, we identified 43 studies evaluating financial incentive programmes for return of service [22]. With the exception of one study from rural South Africa [7], all the reviewed studies evaluate programmes in developed countries (33 studies took place in the United States, five in Japan, two in Canada, and one New Zealand).

While financial-incentive programmes in other countries have not been evaluated in published studies, they have nevertheless been used, for instance in Swaziland [23], Ghana [24], and Mexico [25]. Additional file 1 shows an overview of studies of financial-incentive programme results (i.e. descriptions of outcomes among programme participants), programme effects (i.e. analysis of programme effectiveness at the individual level through comparison of outcomes among participants and non-participants), and programme impacts (i.e. analysis of programme effectiveness at the population level, such as changes in physician density or population mortality) [22]. The table describes the type of study, the type of outcome observed in the study and the main study findings.

While the majority of published studies on financial-incentive programmes examine programmes for doctors [22], a number of articles investigate programmes that enrol other health professionals in addition to doctors, such as nurses, pharmacists or dentists [7,11,26]. Many aspects of the management of a financial-incentive programme are not specific to one type of health worker. In most instances in this article, we thus use the general term "health workers" rather than the name of any specific category of health worker.

Please also note that the definition of medically underserved area varies by country and programme. In general, a medically underserved area is an area where the number of health workers falls below a target. There are, however, many different methods to determine health worker targets, including methods based on need (i.e. the number of health workers necessary to achieve certain population health goals), demand (i.e. the number of health workers sufficient to deliver the health services demanded by patients), or supply (i.e. the number of health workers sufficient to staff existing health care facilities). Commonly, a mix of need, demand and supply criteria is used in the definition of underserved area [22]. In this article, we use the term "medically underserved area" to denote any area that has been identified as a placement site for health workers enrolled in financial-incentive programmes, independent of the particular definition used.

Financial-incentive programmes recruit substantial proportions of participants to underserved areas (the random-effects estimate of the pooled recruitment proportion across the studies in our systematic review was 71% (95% confidence interval 60% to 80%)) [22]. More importantly, a number of studies have found that programme participants are more likely than health workers who do not participate in a financial-incentive programme to remain in underserved areas in the long run [27-30]. While the existing studies have not established causality of programme effects to the exclusion of all other possible explanations for observed difference between programme participants and non-participants [22], overall the evidence to date suggests that financial-incentive programmes can be effective in increasing the supply of health workers to underserved areas (see Additional file 1).

Financial-incentive programmes may be an attractive intervention to place health workers in underserved areas for a number of additional reasons. First, they can subsidize the education of poor students, thus potentially increasing equity of access to higher education. Second, unlike many of the other strategies to attract health workers to underserved areas (such as selective recruitment and training or improvements in working and living conditions [3]), they establish legally enforceable commitments to work in underserved areas and should thus more reliably increase the size of the health workforce in underserved areas. Third, unlike compulsory service policies, they are unlikely to be opposed by health workers.

**Figure 1:**
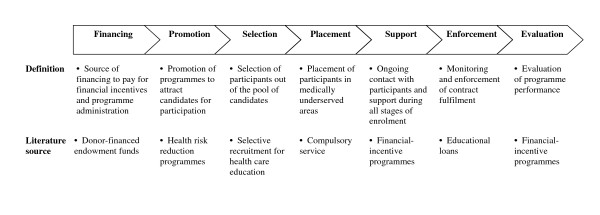
**Management functions of financial-incentive programmes**.

However, financial-incentive programmes are not easy to implement [11,24,31,32]. In this article, we discuss seven management functions essential for the long-term success of financial-incentive programmes (Figure 1). First, programmes need a sustainable source of financing to pay for the financial incentives and programme administration (financing). Next, programmes need to promote their offers in order to attract candidates for participation (promotion), select participants out of the pool of candidates (selection), and place the selected participants in medically underserved areas (placement). Finally, programmes should support the participants during all phases of enrolment (support), enforce the service obligations (enforcement), and evaluate whether programme objectives are achieved (evaluation).

In the following, we describe insights from published studies regarding how these seven management functions can be performed. We draw not only on studies of financial-incentive programmes, but also on initiatives whose objectives or functions partially overlap with those of financial-incentive programmes. For instance, educational-loan programmes share with financial-incentive programmes the objective to recruit participants to receive financial support for education and the management functions of financing, promotion, selection, support, enforcement and evaluation; compulsory service policies share with financial-incentive programmes the objective to increase the supply of qualified workers to certain communities and the management functions of placement, support, enforcement and evaluation.

## The seven management functions

### First function: financing

Four of the five types of financial-incentive programmes shown in Table 1 necessarily require ongoing external financing, while one type (educational loans with service requirement) could theoretically finance itself in the long term if the total amount of money repaid equalled or exceeded programme expenditures. However, such a steady state of revolving refinance will take a long time to achieve, because student loans will start to be repaid only after many years of initial investment [33]. Moreover, both in developed and in developing countries, existing student loan programmes usually require financial injections even in the long term, because losses due to unemployment, default, illness or refusal to repay are usually not priced into the repayment amounts. If they were, such programmes would not be an attractive option for education finance for many eligible students [34]. While substantial long-term finance is thus usually required for the incentive programmes, in many developing countries public finance for such programmes may not be available because governments commonly receive only limited tax revenues, face borrowing constraints, or may not be able to increase the proportion of public finance allocated to spending on education for political reasons [35].

An alternative is to finance the incentive programmes through aid from donors. However, traditional donor financing may not be well-suited for this purpose, which may explain why large international donors rarely support financial-incentive programmes. For one, donors tend to finance projects for periods that may not be sufficiently long to create sustainable programmes and they may be reluctant to provide "running cost" support for training health workers [35]. The latter problem is highlighted by recent discussions about whether large disease-specific aid agencies, e.g. PEPFAR, the Global Fund to Fight AIDS, Tuberculosis and Malaria, and the GAVI Alliance, should invest in human resources for health in developing countries [1,36-38].

In addition, countries that cannot achieve an intended increase in the rate of health worker education through financial-incentive programmes because of limited education capacity may need substantial start-up financing to build educational institutions and to educate health care teachers. The relatively constant flows of traditional donor financing may not allow substantial initial investment with lower rates of continuing finance.

Recent innovation in donor funding may address both shortcomings. On the one hand, donor-financed endowment funds [39] can provide steady long-term money flows well-suited to fund scholarships, loans and salary support. On the other hand, organizations such as the International Finance Facility for Immunisation (IFFIm) [40] can leverage development aid by issuing bonds on international capital markets against long-term commitments of annual payments from donor nations in order to "frontload" aid, allowing immediate large-scale investments (such as in education infrastructure) [41].

In particular situations, countries may be able to increase education rates of health workers through financial-incentive programmes without large start-up investment in additional health care education capacity. For instance, some countries in sub-Saharan Africa, such as Botswana, Lesotho and Swaziland, fund their citizens' health care education in other countries, if the prospective health workers commit to service in their home countries after graduation. However, this strategy may only be feasible for countries with relatively small population sizes and good relationships with countries that have unused health care education capacity. Moreover, the strategy carries the danger that health care workers educated abroad will not return to their country of origin [42]. Financial-incentive programmes could also be used to motivate health workers from relatively well-served countries to practise in underserved countries [43], in which case the latter would benefit from education capacity in the former [44].

Another financing option for financial-incentive programmes would be compensation payments from countries receiving health workers to those countries losing them. It has been argued that developed countries that recruit health workers from African countries with severe health worker shortages have an ethical obligation to compensate the governments of these countries for the loss [45]. While there may be a number of practical problems in implementing compensation payments – for instance, the 2005 *Report of the Global Commission on International Migration *[46] points out that migrating professionals commonly work in more than one country, in which case it is unclear which country is responsible for the payments – financial-incentive programmes seem an especially fitting purpose on which to spend such payments, because they would contribute to decreasing similar losses in the future.

### Second function: promotion

The pool of potential candidates to apply for participation in a financial-incentive programme depends on the start of the programme relative to the stage of health care education (Table 1). In the case of service-requiring scholarships, educational loans with service requirement and service-option loans, potential candidates will be the secondary school graduates who are qualified to pursue a health care education [35]. In the case of loan repayments and direct financial incentives, it will be fully qualified health care professionals who are eligible for participation. The ratio of potential to de facto applicants will depend on the knowledge of the programme among eligible people, as well as the attractiveness of the programme conditions.

There is little published evidence about how secondary school students attain knowledge of tertiary education, including financing options [47-50]. However, a range of communication channels have been successfully used to increase students' knowledge of behaviours to reduce health risks [51]. They include classroom or group sessions led by teachers [52,53] or peers [54,55], or printed material [56]. As postgraduate students and health care professionals commonly use the internet [57-61] and electronic mail [62-64] to obtain and exchange medical information, financial-incentive programmes for fully qualified health workers may be successfully promoted through advertisements on web sites or through email campaigns.

### Third function: selection

Selection of programme participants from among all candidates who apply for a place in a financial-incentive programme can contribute to achieving the main objective of the programme, i.e. to increase the supply of health workers to medically underserved areas, as well as supplementary policy goals. One strategy to maximize the effectiveness of the programme in increasing the supply of health workers to underserved areas is to select candidates based on characteristics observed to be associated with a low probability of defaulting on the service obligation and a high probability of remaining in an underserved area after completion of the obligation.

There is evidence from both developing countries [65-67] and developed countries [65,68-72] that health care graduates from rural background are more likely to choose rural practice than their peers from urban areas. For instance, a 2003 study in South Africa found that 10 years after graduating from medical school, doctors of rural origin were 3.5 times more likely than doctors of urban origin to practise in rural areas [67].

In settings where the selected students would have attained a health care degree even if they had not received a financial incentive, selection on factors associated with the propensity to work in underserved areas will not contribute to programme effectiveness – unless programme participation has an effect on the propensity to work and remain in underserved areas and this effect differs by the selection factors. However, in settings where without the financial support from a programme large proportions of the selected students would have been unable to finance a health care education, selective recruitment is likely to improve programme effectiveness because it can change the composition of health care students, such that the average propensity for underserved practise increases.

Policy-makers can also use selection into a financial-incentive programme to achieve supplementary health care education goals. For instance, financial equity of access to tertiary education can be improved by basing eligibility for the financial incentive on means tests [73]. Merit can be rewarded by basing eligibility on secondary school performance. The proportion of students from groups who are traditionally underrepresented in health care education can be increased if financial incentives are preferentially given to members of these groups.

### Fourth function: placement

Placement of programme participants in particular underserved areas is likely to be an important determinant of programme success. Policy-makers first need to agree on a definition of "medically underserved area". Some programmes in developed countries have used simple definitions of "medically underserved areas" (e.g. rural communities with populations of 5000 or less [74] or towns or villages with populations of 2500 or less [10]), while others have used a range of need, demand, and supply criteria (e.g. population health, demographic and socioeconomic characteristics of the population, and current health worker-to-population ratios [75,76]) to identify areas.

Once areas have been designated as "medically underserved", the programme participants need to be matched to underserved areas. In order to maximize the social value of financial-incentive programmes, policy-makers could consider placing participants preferentially in those underserved areas where unmet health care need is greatest, because the impact of a placement on population health in these areas is likely to be most significant.

Without such a preferential placement policy, it is possible that the neediest populations will benefit least from financial-incentive programmes. For instance, a study of the National Health Service Corps (NHSC), a national financial-incentive programme that has operated in the United States since 1972 [77], found that the poorer an underserved area eligible to receive an NHSC physician and the worse its population health, the less likely it was to receive a physician through the programme [78].

However, such a policy would strongly restrict participants' choice of placement area. As a result, participants may be less likely to be satisfied with their work and personal life during the obligated service, decreasing the chances of long-term retention in the placement area. A study of the NHSC found that NHSC enrolees "placed in rural sites in the late 1980s experienced a site-matching process that they felt offered few acceptable sites" and "offered little opportunity to locate the best-suited site among those offered" [32]. A study from South Africa concluded that physicians were dissatisfied with their placements for compulsory community service because they were forced to serve in a particular location and because they felt that the placement disrupted their social lives [79] – two problems that should be less likely to occur if programme participants were given the choice to serve in one of many underserved areas.

A number of studies in the United States found that programme participants were significantly less likely to remain in the same underserved area over time than health workers who worked in underserved areas but had not participated in any financial-incentive programme [12,27,32,80]. However, several other studies in the United States found that participants in financial-incentive programmes are more likely to continue to practice in some underserved area [27,32] or to provide care to an underserved population [28,30,32] than health workers who had chosen – without financial incentive – to start practising in an underserved area at the same time that programme participants started serving their obligations.

These findings can be explained as follows. Participants in financial-incentive programmes are more likely to practise in underserved areas in the long run than those non-participants who at some point in time choose to work in an underserved area. However, participants are not placed in their preferred underserved area. Thus, participants relocate from the placement area to their preferred underserved area after having completed their service obligation.

Financial-incentive programmes aiming to attain high retention of obligated health workers in the placement area should attempt to accommodate health workers' wishes to practise in particular underserved areas as far as possible. Optimal placement could be achieved, for instance, by a matching process such as the one used for specialist training places in the United States, whereby candidates and training institutions rank each other in order of declining preference and a computer algorithm implements explicit rules to identify the best assignment of candidates to institutions [81].

### Fifth function: support

It is likely that the satisfaction of health workers with their participation in financial-incentive programmes will be important in determining whether they start and complete their service obligations and whether they remain in an underserved area in the long run [80]. Evidence from the United States shows that participants' work and life satisfaction can vary substantially by programme type [12,80]. Such differences across programmes can be due to a number of reasons.

Different types of health workers may choose to participate in different programmes, and programmes may differ in how far they take participants' wishes into account in selecting placement areas (see above). However, programmes may also be able to influence participants' satisfaction before and during their time of service by offering support. For instance, the NHSC has developed "tools to prepare providers for underserved areas", which include learning modules on "personal and professional development", "cross cultural issues in primary care", "leading group discussions", and health care issues important in working with "disenfranchised populations" (such as adolescent pregnancy, HIV/AIDS, child abuse, domestic violence or substance abuse) [82]. In addition, the NHSC has established a "recruitment, training and support center" which maintains contact with underserved areas, offers "guidance and support to NHSC scholars during the relocation process" and monitors participants during their service [83].

The Friends of Mosvold Scholarship Scheme (FOMSS), which provides scholarships to health care students from the rural Umkhanyakude district of South Africa in return for a commitment to work in the district after graduation [84], assigns each participant a mentor. The mentor supports the participant during her studies: "Regular visits to the campuses supplemented by telephone calls by the main mentor made the students feel that he was there for them and that he cared. Struggling students were encouraged to analyse their situation using questions such as 'What do you think is the problem?' and 'What have you done to find a solution?'. Wherever practicable, solutions were found quickly and included interventions such as the student (and sometimes the mentor) contacting a lecturer or head of department, finding better accommodation, or providing a computer for FOMSS students where university resources were inadequate, etc." [7].

As described for FOMSS, ongoing contact with participants enables managers of financial-incentive programmes to detect difficulties that health workers are facing and to intervene speedily. In addition to assigning participants to mentors, programmes can ensure that they remain in close contact with participants through regular meetings with individual health workers, discussions with groups of participants, telephone hotlines [83], or frequent surveys of participant satisfaction [19,85,86]. Programmes can offer support by initiating peer group meetings [79], establishing peer-support systems, such as Balint groups [87], paying for education courses that teach skills relevant to health care in underserved areas [88], or funding equipment that participants need in their clinical work.

### Sixth function: enforcement

Programme participants can default on their obligation in several different ways. In programmes without a repayment or buy-out option, they can, firstly, refuse placement and service after having received the financial incentive and, secondly, comply with the placement but fail to perform the specific duties they are obliged to perform in the placement area. An example of the latter type of default is a physician who fails to fulfil her obligation to work in a public-sector hospital in the placement area and instead sees patients in private practice. While the first type of default is comparatively easy to detect (for instance, through spot checks or calls to local hospital administrators), the second type can be difficult to detect (for instance, if the health services administration in the placement area is weak). In programmes with a buy-out option, participants default if they neither fulfil their service obligation nor repay the financial incentive.

In order to ensure that participants fulfil their obligations, programmes must have a monitoring strategy in place to identify defaulters, as well as a strategy to deal with detected defaulters. The strategies will depend on legal, institutional and technological factors specific to a country. Experiences from educational-loan programmes in Africa suggest that rather than building up an infrastructure to monitor defaults on service or financial obligations themselves, financial-incentive programmes should outsource this function to existing institutions that already have the structures and experience to deal with contractual default, such as the tax system, the social security system or banks [73].

An alternative to using such large existing systems to monitor participants is community-based monitoring approaches [89], including monitoring through local leaders, citizen report cards ("participatory surveys that provide quantitative feedback on user perceptions on the quality, adequacy and efficiency of public services", i.e. the services of health workers participating in financial-incentive programmes [90]), or community score cards ("qualitative monitoring tools that are used for local level monitoring and performance evaluation of services" [90]). Community-based monitoring may be preferable for relatively small local financial-incentive programmes.

Monitoring and punishment are reactive approaches to reduce default. Preventive strategies to decrease default rates include regulation, such as withholding diplomas or licenses from scholarship recipients until they have completed their service [35], requiring completion of the obligated service for specialist training [66], or restricting the visa eligibility of obligated health workers before completion of their service [15].

### Seventh function: evaluation

A large number of descriptive case studies and cohort studies have evaluated financial-incentive programmes (Additional file 1) [22]. However, with one exception from South Africa [7], all of the published evaluations have taken place in industrialized countries. In order to improve the scope of the existing evidence, financial-incentive programmes in developing countries should collect quantitative and qualitative data on their experiences and outcomes and publish them.

While the evidence on the effects of financial-incentive programmes on recruitment and long-term retention in underserved areas is extensive, it has a number of limitations. For one, the evidence may not be generalizable to many of the countries that suffer from the most severe shortages of health workers in rural and remote areas, in particular sub-Saharan African countries. The majority of published evaluations of financial-incentive programmes took place in the United States (Additional file 1). The United States health care education system, however, is unusual in comparison to many other countries in that students pay a high tuition for their education. Within the United States, it has been found that medical students' propensity to enrol in a financial-incentive programme increases with their debt burden [32]. Thus, it would seem plausible that in countries where health care education is subsidized to such an extent that students have to pay very little tuition, financial-incentive programmes could be substantially less attractive than in the United States.

However, in a number of countries with very low tuition for health care education, students nevertheless incur substantial expenses, for instance, for housing, meals, medical textbooks and equipment [91], requiring them to seek funding support, for instance, through a financial-incentive programme. Future studies should evaluate outcomes of financial-incentive programmes in developing countries, such as Swaziland [23], Ghana [24], and Mexico [25].

Another fundamental difference between the United States and many of the developing countries that currently face severe health worker shortages is that the income differential between underserved and well-served areas is larger in the latter than in the former. Pathman and colleagues find that United States physicians fulfilling a service commitment in underserved areas did not earn significantly less than physicians without such an obligation [32].

In contrast, in many developing countries health workers in private practice earn substantially more than their colleagues in the public sector, and opportunities for full-time or part-time work in private practice may exist only in well-served urban areas and not in rural and remote areas where financial-incentive programmes offer positions. Insofar as financial incentives simply compensate for income differentials between underserved and well-served areas, they are unlikely to be attractive. Salary mark-ups specifically for participants in financial-incentive programmes, on the other hand, may not be feasible because they would imply that participants earn more than non-participants working in underserved areas, which may be difficult to justify.

Thus, in some developing countries, financial incentive programmes similar to the ones offered in the United States and other developed countries may not be successful unless the incomes of all health workers in underserved areas are increased. An example of such a universal change in salary structures is the "rural allowance" in South Africa, which was added in 2004 to the salaries of public-sector health workers in rural areas [92]. In some countries, work in underserved areas would be financially more attractive if health workers were allowed to rotate between the public sector in underserved areas and the private sector in well-served areas.

Another limitation of the evidence is that it is exclusively based on observational studies, which do not allow to firmly establish a causal relationship between programme participation and work in underserved areas. It seems plausible that financial-incentive programmes place health workers in underserved areas who would never have worked in such areas.

Furthermore, financial-incentive programmes may expose participants who would have worked in an underserved area without financial incentive to experiences that they would not have had, had they not enrolled. Such programme-specific experiences (e.g. preparation for work in the underserved area and mentoring during the obligated service) could increase participants' propensity to work in underserved areas in the long run.

While such effects are plausible, it is difficult to rule out the possibility that those workers who choose to participate would have practised in underserved areas for exactly the same length of time (or even longer) without a financial incentive. In order to strengthen the evidence on the effects of financial-incentive programmes, researchers should conduct controlled experiments, wherever funders and policy-makers are willing to support such studies.

## Comparison of financial-incentive programmes with other interventions to increase the supply of health workers in underserved areas

Financial-incentive programmes are only one type of intervention to increase the supply of health workers in underserved areas. Two other types are compulsory service and non-financial incentives. In the following, we will briefly describe these two types of alternative interventions and contrast them with financial-incentive programmes.

### Compulsory service versus financial-incentive programmes

Compulsory service policies require health workers (e.g. all doctors or all nurses) who are educated in a country to work for a period of time in an underserved area in that country. Such programmes have been established in many countries worldwide.

Beginning in the 1920s, the Soviet Union required all medical, dental and nursing graduates to serve for three years in rural areas [93]. In 1936, Mexico started requiring six months of rural service as a condition for medical students to graduate from medical school. The six-month requirement was later extended to one year [94]. Other countries in Latin America followed with similar programmes, including Cuba (in 1960) [95], the Dominican Republic (in the 1960s) [96,97], Ecuador (in 1970) [94], and Bolivia (in 1979) [94]. In Africa, Nigeria established a National Youth Service Corps in 1973, which requires all graduates of tertiary education institutions, including health workers, to work for one year in an underserved area [98]. Since 1998, all South African medical graduates have to perform a one-year "compulsory community service" [79]. Compulsory service policies also exist in South Asia (e.g. in several states of India [99,100]), the Eastern Mediterranean (e.g. Iraq [101]) and Europe (e.g. Greece [102]).

While compulsory service is used widely, the evidence on its performance is scarce. The 2007 United States Council on Graduate Medical Education Report *New Paradigms for Physician Training for Improving Access to Health Care *comes to the conclusion that "[t]he impact of these [compulsory service] programmes had been difficult to assess, and there is a dearth of rigorous studies of their effectiveness and viability. It is clear from existing information that it is possible to create and sustain such programmes over a period of decades, although not necessarily with enthusiastic support of those required to serve" [95].

The evidence that does exist is mainly on the satisfaction of health workers with their compulsory service. An evaluation of the South African compulsory community service finds that 64% of the doctors felt that "they had developed professionally" during the service, but that their development had taken place mostly "in the area of gaining confidence and insight in themselves as practitioners, as opposed to formal learning of clinical skills from supervisors" [79]. Similarly, a study in Ecuador reports that 94% of health workers found "their [compulsory] year of rural service rewarding both personally and professionally" [94]. Many of the participants "commented on how much they learned about doctor-patient relations" and "[s]ome said they matured emotionally, learned the meaning of responsibility, and acquired greater self-confidence" [94].

Because very few empirical studies have been published on compulsory service, a comparison of the programmes to financial-incentive programmes has to be based on theoretical considerations. Table 2 outlines differences in the characteristics and possible effects between the two types of interventions.

**Table 2:** Comparison of financial-incentive programmes to compulsory service

	**Financial-incentive programmes**	**Compulsory service**
Enrolment	Self-selected	Universal

Compulsion	No	Yes

Length of service	Commonly >3 years	Commonly 1–3 years

Effect on equity of access to tertiary education	Improvement possible	None

Effect on total number of health workers	Increase possible	Decrease possible

Effect on composition of health worker population	Increase in proportion of health workers from poor backgrounds possible	Increase in proportion of lower-quality health workers possible

The main difference is, of course, that compulsory service policies force all health workers (in a particular category) to serve, while financial-incentive programmes enrol only those health workers who choose to participate. Thus, compulsory service policies (if they can be enforced) ensure that a substantial proportion of workers who – given the choice – would never have practised in underserved areas do so for some period and that, at least in the short term, such requirements will be effective in increasing the supply of health workers to underserved areas. In contrast, financial-incentive programmes cannot ensure that they will be effective in recruiting health workers to underserved areas who would not have chosen to do so without financial incentive.

Compulsion, however, implies a "loss of autonomy" and can create an "aversion", which may lead to a number of negative consequences [95]. For one, the introduction of compulsory service may be difficult politically. For instance, in 2008, a strike of medical students and doctors forced the government of Kerala, India, to reduce the planned compulsory rural service for doctors from three years to one year [103,104]. Further, it is possible that health workers who are forced to work in an underserved area for some period are less likely to voluntarily work in such an area and more likely to emigrate to another country in the long run.

Moreover, compulsory service may decrease the attractiveness of a health care education because it limits graduates' choices of where to work. As such, compulsory service could lead to fewer applicants to health care education institutions, which could reduce the total number of health workers educated per time (if the number of education places exceeds the number of qualified applicants) [35], or decrease the average quality of health care students (if education institutions lower entry requirements in order to fill their education places) [95]. In contrast, financial-incentive programmes could increase the total number of educated health workers and increase the proportion of students from poor backgrounds, if the financial incentives enable students who would otherwise not have been able to do so to pay for a health care education, and if a country's education system can absorb the additional students.

### Non-financial incentives versus financial incentives

Health workers are motivated not only by financial compensation but also by other factors, such as altruism, the satisfaction of successfully applying their skills in caring for their patients and recognition from their peers. For instance, a study in Benin and Kenya found in semistructured interviews that nurses and doctors more commonly referred to "healing patients", "vocation", "professional satisfaction" and "recognition by supervisors" than to "remuneration" when asked what currently encourages them to do their work well [105].

A study in rural Viet Nam found that "the main motivating factors for health workers were appreciation by managers, colleagues and the community, a stable job and income and training", while "the main discouraging factors were related to low salaries and difficult working conditions" [106]. As such, non-financial factors should be expected to influence the supply of health workers in underserved areas.

A WHO study found that while health workers in the public sector in Cameroon, Ghana, South Africa, Uganda and Zimbabwe most commonly considered "salaries" as one of the "key issues ... that will motivate them to remain in the country" (between 68% and 85% of the respondents in the five countries), they also considered non-financial factors to be important in their migration decisions – for instance, the "working environment" (between 36% and 81%) and "opportunities for education and training" (between 29% and 67%) [107].

In addition to such work-related factors, living conditions are likely to be important in determining health workers' decisions to move to and remain in underserved areas. In Ecuador, health workers fulfilling their compulsory service ranked transportation "highest as an adaptation problem, followed by, in descending order, communication, housing, food, and access to potable water and electrical power" [94].

In the United States, physicians working in the Navajo Area India Health Services referred to "the poor local school system" and "marginal housing facilities" as reasons why they might leave their positions [108]. Rural doctors in Limpopo, a poor rural province of South Africa, provided a range of themes in response to the question about which interventions they thought would retain South African doctors in rural hospital service in the province, including financial incentives ("increasing salaries and rural allowances"), improvements in working conditions (such as "ensuring career progression", "providing continuing medical education", "improving the physical hospital infrastructure and rural referral systems", "ensuring the availability of essential medical equipment and medicines", and "strengthening rural hospital management"), and improvements in living conditions (such as "improving rural hospital accommodation", "providing recreational facilities", and "assisting rural doctors' families") [19].

Work-related factors that affect health workers' location choices can potentially be influenced through investment in health care facilities, medical equipment and workplace safety [35], as well as through a range of management interventions [109,110], such as training of supervisors [35], "quality improvement teams", "team building", "participatory problem assessments and problem-solving processes", and "development of career development plans" [105]. Living conditions can be improved through investment in infrastructure in underserved areas, such as roads, electricity, telecommunication, water, sanitation and housing. However, only a few countries (such as Thailand [111] and Zambia [112]) have implemented interventions to improve health workers' working or living conditions in underserved areas, and evidence on their effectiveness in increasing the supply of health workers in those areas is largely lacking [3,113].

In thinking about alternative interventions to increase the supply of health workers in underserved areas, governments and donors should bear in mind that such interventions are usually not mutually exclusive. For instance, in South Africa the national compulsory community service [79] operates alongside national [92] and local [7] financial-incentive programmes. Non-financial incentives improving health workers' satisfaction with their professional and personal lives could be important in improving long-term retention of health workers in areas to which they were originally attracted by a financial incentive [22]. Zambia established a "Health Workers Retention Scheme" to improve the supply of doctors to "rural and underserved parts of Zambia". The scheme provides a financial incentive (a "rural hardship allowance") and several non-financial incentives, including guaranteed eligibility for postgraduate training after three years of service and investment to improve housing for health workers in underserved areas [112].

Policy-makers should further consider that on the continuum from incentive to compulsion there are intermediate forms of interventions, which may be the best choices in particular situations. For instance, in some countries practice in underserved area is not compulsory but necessary or desirable for acceptance into specialist training programmes [114]. Incentives, on the other hand, can come in the form of cash payments to the health worker, earmarked allowances for housing or schooling, fringe benefits (such as old-age pension or health insurance), and improvements in living and working conditions in underserved areas [35].

## Conclusion

Financial-incentive programmes for return of medical service in underserved areas have been used in both developed and developing countries. The existing literature on financial-incentive programmes and related interventions suggests a number of ways how the seven management functions that are essential for the long-term success of financial-incentive programmes can be successfully implemented:

• financing (programmes may benefit from innovative donor financing schemes, such endowment funds, international financing facilities or compensation payments);

• promotion (programmes should use tested communication channels in order to reach secondary school graduates and health workers);

• selection (programmes may use selection criteria to ensure programme success and to achieve supplementary policy goals);

• placement (programmes may use matching of participants to areas to ensure programme success);

• support (programmes should prepare participants for their time in an underserved area, stay in close contact with participants throughout the different phases of enrolment and help participants by assigning them mentors, establishing peer support systems or financing education courses relevant to work in underserved areas);

• enforcement (programmes may use community-based monitoring or outsource enforcement to existing institutions);

• evaluation (in order to increase the scope of evidence on the effectiveness of financial incentives in supplying health workers to underserved areas, programmes in developing countries should evaluate their performance; in order to improve the strength of the evidence on the effectiveness of financial incentives, controlled experiments should be conducted where feasible).

Financial-incentive programmes have a number of advantages and disadvantages in comparison with other interventions to increase the supply of health workers to medically underserved areas. Unlike non-financial incentives, they establish legally enforceable commitments to work in underserved areas; however, they may not improve the working or living conditions in underserved areas, which are important determinants of health workers' long-term retention in those areas. Unlike compulsory service policies, they will not be opposed by health workers; however, they cannot guarantee that they supply health workers to underserved areas who would not have worked in such areas without financial incentives. Financial incentives, non-financial incentives, and compulsory service are not mutually exclusive and may positively affect each other's performance.

## Competing interests

The authors declare that they have no competing interests.

## Authors' contributions

TB and DEB jointly conceived the study and contributed equally to the analyses and to the drafting and revising of the manuscript. Both authors have approved the final version of the manuscript.

## Authors' information

In addition to his email address at the Harvard School of Public Health, TB can be reached at his email address at the Africa Centre for Health and Population Studies: tbarnighausen@africacentre.ac.za.

## Supplementary Material

Additional file 1 **Overview of evidence on financial-incentive programmes for return of medical service**. Table in landscape format exceeding one A4 page in length. Click here for file
