# Supplementary material for: Designing financial-incentive programmes for return of medical service in underserved areas: seven management functions
Source: Hum Resour Health. 2009 Jun 26;7:52. doi: 10.1186/1478-4491-7-52 (PMC2714830; doi:10.1186/1478-4491-7-52)
Supplement: Additional file 1 — Overview of evidence on financial-incentive programmes for return of medical service. Table in landscape format exceeding one A4 page in length. [file 1478-4491-7-52-S1.doc]

**Appendix**

**Table A1: Overview of evidence on financial-incentive programmes for return of medical service**

| **Study** | **Programme** | **Country** | **Type of study** | **Type of outcome** | **Conclusions** |
| --- | --- | --- | --- | --- | --- |
| Fitz et al. 1977 [1] | Commonwealth Fund Medical Under-graduate Scholarship Program | USA | Description of programme outcomes | *Programme results*  Recruitment  Retention | 54% of all participants fulfilled their service obligation and 4% repaid the financial incentive.  51% of all participants practiced in small communities for most of their working lives. |
| Mason 1971 [2] | State scholarship and educational loan programmes | USA | Description of programme outcomes | *Programme results*  Recruitment  Retention | 60% of participants fulfilled their obligation to practice in an underserved area, 37% repaid the financial incentive.  Across programmes, between 50% and 90% of participants remained in rural communities after having fulfilled their obligation. |
| Bradbury 1963 [3] | Carolina Rural Loan Program | USA | Description of programme outcomes | *Program results*  Recruitment  Retention  Participant satisfaction | 75% of participants fulfilled their obligation to practice in an underserved area.  71% of participants in the financial-incentive programme were satisfied with their overall experience. |
| Navin and Nichols 1977 [4] | Arizona Medical Student Exchange Program | USA | Description of programme outcomes  Time series | *Programme results*  Recruitment  Retention  *Programme impact*  Health system | 59% of participants fulfilled their obligation to practice in an underserved area, while 37% of participants repaid the financial incentive  85% of participants who fulfilled their obligation remained in Arizona.  The programme did not succeed in increasing the medical student population density in Arizona. |
| Bass and Copeman 1975 [5] | Ontario Under-serviced Area Program | USA | Description of programme outcomes  Time series | *Programme results*  Recruitment  Retention  *Programme impact*  Health system | 53% of participants fulfilled their obligation to practice in an underserved area, while 47% repaid the financial incentive.  74% of participants who fulfilled their obligation remained at the original placement location.  The programme was effective in increasing the number of physicians practicing in small communities in northern Ontario. |
| Anderson and Rosenberg 1990 [6] | Ontario Under-serviced Area Program | USA | Before-after comparison | *Programme impact*  Health system | Increase in supply of physicians to underserved areas cannot be attributed to the programme. |
| Inoue et al. 1997 [7] | Jichi Medical University | Japan | Description of programme outcomes | *Programme results*  Recruitment  Retention | 96% of all participants fulfilled their obligation to practice in an underserved area, while 4% repaid the financial incentive.  67% of participants remained in the prefecture of original placement after having fulfilled their obligation. |
| Inoue et al. 2007 [8] | Jichi Medical University | Japan | Description of programme outcomes | *Programme result*  Recruitment  *Programme effect*  Provision of care | 98% of participants fulfilled their obligation to practice in an underserved area.  Participants were more likely than non-participants to practice in a rural area. |
| Matsumoto et al. 2008 [9] | Jichi Medical University | Japan | Retrospective cohort study | *Programme effect*  Provision of care | After having fulfilled their obligation to practice in an underserved area, participants were about four times more likely to work in rural areas than non-participants. |
| Matsumoto et al. 2008 [10] | Jichi Medical University | Japan | Retrospective cohort study | *Programme results*  Retention | 21% of participants of rural background, and only 12% of participants of urban background, remained in a rural area after having fulfilled their service obligation. |
| Matsumoto et al. 2008 [11] | Jichi Medical University | Japan | Description of programme outcomes | *Programme results*  Recruitment  Retention | 95% of participants fulfilled their obligation to practice in an underserved area.  Of all participants who had fulfilled their obligation at least 6 years ago 70% remained in the prefecture of original placement. |
| Woolf et al. 1981 [12] | National Health Service Corps | USA | Comparison of characteristics of underserved areas with and without programme participants  Discriminant analysis | *Programme impact*  Health system | Underserved communities that had less resources and higher need for health care were less likely to receive programme participants than underserved communities that were better-off. |
| Stamps and Kuriger 1983 [13] | National Health Service Corps | USA | Descriptive study | *Programme result*  Retention | 56% of the participants who were currently fulfilling their obligation intended to practice in a rural area after fulfilling their obligation. |
| Stone et al. 1991 [14] and Brown et al. 1990 [15] | National Health Service Corps | USA | Descriptive study | *Programme results*  Retention  Participant satisfaction  Family satisfaction | 67% of participants who were currently fulfilling their practice obligation intended to remain in their placement site after fulfilling the obligation.  Reasons for intending to leave the placement site included dissatisfaction with the community, the salary, and the workload, as well as unmet needs of family members. |
| Pathman et al. 1992 [16] | National Health Service Corps | USA | Retrospective cohort study | *Programme effect*  Retention | Participants were about twice as likely as non-participants to leave their practice of original placement and about 50% more likely to leave rural practice than non-participants. |
| Pathman et al. 1994 [17] | National Health Service Corps | USA | Retrospective cohort study | *Programme effects*  Retention | Participants were about half as likely as non-participants to remain in a non-metropolitan area and about three times less likely than non-participants to remain in the same practice. |
| Pathman et al. 1994 [18] | National Health Service Corps | USA | Retrospective cohort study | *Programme result*  Participant satisfaction  *Programme effects*  Retention  Participant satisfaction | Five years after starting work at a practice site, participants were less than half as likely as non-participants to have remained at the site.  Participants were less satisfied with their work and personal lives in the underserved area than non-participants. |
| Pathman and Konrad 1996 [19] | National Health Service Corps | USA | Retrospective cohort study | *Programme results*  Retention  Participant satisfaction  Family satisfaction | Minority and non-minority participants did not differ in their retention in the practice of original placement after having fulfilled their service obligation.  Minority physicians reported lower satisfaction with their work and personal lives in the underserved area (for themselves and their families) than non-minority physicians. |
| Rosenblatt et al. 1996 [20] | National Health Service Corps | USA | Description of programme outcomes | *Programme results*  Retention  Participant satisfaction | Six years after having fulfilled their practice obligation 25% of participants continued to practice in the county of original placement, while 27% had left the original placement site to practice in another rural county.  33% of participants rated their experience in the programme as “positive”. |
| Cullen et al. 1997 [21] | National Health Service Corps | USA | Description of programme outcomes | *Programme result*  Retention | 8-10 years after having graduated from medical school, 20% of the participants remained in the county of their original placement, while 40% remained in a rural county. 11-13 years after graduation these proportions had fallen to 17% and 36%, respectively. 14-16 years after graduation they had fallen to 13% and 35%. |
| Xu et al. 1997 [22] | National Health Service Corps | USA | Retrospective cohort study | *Programme effect*  Provision of care | Participants were significantly more likely to practice in an underserved area ten years after graduating from medical school than non-participants. |
| Xu et al. 1997 [23] | National Health Service Corps | USA | Retrospective cohort study | *Programme effect*  Provision of care | 30% of participants’ patients were either considered poor or had Medicaid as their primary insurance but only 19% of non-participants’ patients had these characteristics. |
| Singer et al. 1998 [24] | National Health Service Corps | USA | Retrospective cohort study | *Programme effect*  Retention | After five years of work in a community health centre, 36% of participants, but only 17% of non-participants, still worked in the same centre. |
| Rabinowitz et al. 2000 [25] | National Health Service Corps | USA | Retrospective cohort study | *Programme effect*  Provision of care | “Participation in the NHSC is the only experiential factor related to caring for the underserved”. |
| Rabinowitz et al. 2001 [26] | National Health Service Corps | USA | Retrospective cohort study | *Programme effect*  Provision of care | Participants in the NHSC were significantly more likely than non-participants to provide primary care in a rural area. |
| Mofidi et al. 2002 [27] | National Health Service Corps | USA | Description of programme outcomes | *Programme result*  Retention | 47% of participants continued to provide care to the underserved after their obligated service. |
| Brooks et al. 2003 [28] | National Health Service Corps | USA | Retrospective cohort study | *Programme effect*  Provision of care | 13% of rural primary care physicians, but only 3% of suburban and 3% of urban primary care physicians, had participated in the programme. |
| Porterfield et al. 2003 [29] | National Health Service Corps | USA | Descriptive study | *Programme result*  Retention | 7 to 17 years after starting to fulfil their practice obligation, 53% of the participants still worked in an underserved area. |
| Probst et al. 2003 [30] | National Health Service Corps | USA | Retrospective cohort study | *Programme effect*  Provision of care | 28% of the patients discharged by programme alumni were Medicaid patients, while only 19% of the patients discharged by non-alumni were Medicaid patients. |
| Holmes 2004 [31] | National Health Service Corps | USA | Retrospective cohort study | *Programme effects*  Provision of care  Retention | Participants were less likely to remain at their first practice location than non-participants.  Participants were more likely to serve in any underserved area than non-participants. |
| Pathman et al. 2005 [32] | National Health Service Corps | USA | Pre-post comparison | *Programme impact*  Health | The programme may have contributed to improvements in age-adjusted mortality rates in underserved communities, in particular in communities that received programme participants for more than 11 years. |
| Holmes 2005 [33] | National Health Service Corps | USA | Retrospective cohort study | *Programme impact*  Health system | The programme contributed 10-11% to the existing US physician workforce in underserved areas. |
| Pathman et al. 2006 [34] | National Health Service Corps | USA | Retrospective cohort study | *Programme impact*  Health system | Presence of a programme participant increased the supply of non-participating physicians in underserved areas on average by 6%. |
| Rittenhouse et al. 2008 [35] | National Health Service Corps | USA | Retrospective cohort study | *Programme effect*  Provision of care | Participants were significantly more likely to work in a community health centre than non-participating physicians. |
| Weiss et al. 1980 [36] | Scholarship for Indian students in health sciences | USA | Description of programme outcomes | *Programme result*  Recruitment | In a programme in which participants are not obligated to serve in an underserved area, 74% of participants decided to work in an underserved area. |
| Holmes and Miller 1985 [37] | Oklahoma Rural Medical Education Scholarship Loan | USA | Description of programme outcomes | *Programme result*  Recruitment | 68% of participants fulfilled their practice obligation, while 32% repaid the financial incentive. |
| Lapolla et al. 2004 [38] | Oklahoma Rural Medical Education Scholarship Loan | USA | Description of programme outcomes | *Programme results*  Recruitment  Retention | 75% of participants fulfilled their obligation to practice in an underserved area, while 25% repaid the financial incentive.  53% participants remained in the placement community after having fulfilled their obligation. |
| Pathman et al. 2000 [39] | National Health Service Corps  Indian Health Service Corps  State scholarships  State loan repayment programmes  Practice and hospital-sponsored financial incentives | USA | Retrospective cohort study | *Programme effect:*  Provision of care | In comparison to non-participants, participants in financial- incentive programmes were about five times more likely to practice in rural areas and 85% more likely to care for underserved populations. |
| Dunbabin et al. 2006 [40] | New South Wales Department of Health Rural Resident Medical Officer Program | Australia | Description of programme outcomes | *Programme results:*  Recruitment  Retention | About 87% of participants fulfilled their obligation to practice in a rural area.  Retention in rural communities after completion of the obligation was substantial. |
| Jackson et al. 2003 [41] | West Virginia Community Scholarship Program  West Virginia Health Sciences Scholarship Program  West Virginia Recruitment and Retention Community Program  West Virginia State Loan Repayment Program | USA | Retrospective cohort study | *Programme results:*  Recruitment  Participant satisfaction  *Programme effects:*  Retention  Participant satisfaction | 78% of participants fulfilled their obligation to practice in an underserved area.  Retention in the first practice site was not significantly different between programme participants and non-participants.  98% of programme participants, but only 85% of non-participants, “agreed that clinical worker was personally rewarding”. |
| Pathman et al. 2004 [42] | State scholarship programmes  State loan programmes with service option  State loan repayment programmes  State direct financial- incentive programmes for medial residents  State direct financial- incentive programmes for fully trained health professionals | USA | Description of programme outcomes  Retrospective cohort study | *Programme results:*  Participant satisfaction  Family satisfaction  *Programme effect:*  Retention | Participants in programmes that enrolled physicians after graduation from medical school were more likely to fulfil their service obligation than participants in programmes that enrolled participants during medical school.  Participants were about 25% less likely to remain at their site of first practice than non-participants.  The majority of participants in financial-incentive programmes were satisfied with their experience; their spouses were significantly less satisfied. |
| Ross 2007 [43] | Friends of Mosvold Scholarship Scheme | South Africa | Description of programme outcomes | *Programme result:*  Recruitment | All participants fulfilled their obligation to practice in the underserved area. |

**References**

1. Fitz RH, Mawardi BH, Wilber J: **Scholarships for rural medicine. The Commonwealth Fund experience with a pre-World War II indenture program.** *Trans Am Clin Climatol Assoc* 1977, **88:**191-196.

2. Mason HR: **Effectiveness of student aid programs tied to a service commitment.** *J Med Educ* 1971, **46:**575-583.

3. Bradbury SF: **The North Carolina Medical Care Commission: evaluation of the Rural Loan Program by recipients of medical and dental loans.** *N C Med J* 1963, **24:**489-491.

4. Navin TR, Nichols AW: **Evaluation of the Arizona Medical Student Exchange Program.** *J Med Educ* 1977, **52:**817-823.

5. Bass M, Copeman WJ: **An Ontario solution to medically underserviced areas: evaluation of an ongoing program.** *Canadian Medical Journal* 1975, **113:**403-407.

6. Anderson M, Rosenberg MW: **Ontario's underserviced area program revisited: an indirect analysis.** *Soc Sci Med* 1990, **30:**35-44.

7. Inoue K, Hirayama Y, Igarashi M: **A medical school for rural areas.** *Med Educ* 1997, **31:**430-434.

8. Inoue K, Matsumoto M, Sawada T: **Evaluation of a medical school for rural doctors.** *J Rural Health* 2007, **23:**183-187.

9. Matsumoto M, Inoue K, Kajii E: **A contract-based training system for rural physicians: follow-up of Jichi Medical University graduates (1978-2006).** *J Rural Health* 2008, **24:**360-368.

10. Matsumoto M, Inoue K, Kajii E: **Characteristics of medical students with rural origin: implications for selective admission policies.** *Health Policy* 2008, **87:**194-202.

11. Matsumoto M, Inoue K, Kajii E: **Long-term effect of the home prefecture recruiting scheme of Jichi Medical University, Japan.** *Rural Remote Health* 2008, **8:**930.

12. Woolf MA, Uchill VL, Jacoby I: **Demographic factors associated with physician staffing in rural areas: the experience of the National Health Service Corps.** *Med Care* 1981, **19:**444-451.

13. Stamps PL, Kuriger FH: **Location decisions of National Health Service Corps physicians.** *Am J Public Health* 1983, **73:**906-908.

14. Stone VE, Brown J, Sidel VW: **Decreasing the field strength of the National Health Service Corps: will access to care suffer?** *J Health Care Poor Underserved* 1991, **2:**347-358.

15. Brown J, Stone V, Sidel VW: **Decline in NHSC physicians threatens patient care.** *Am J Public Health* 1990, **80:**1395-1396.

16. Pathman DE, Konrad TR, Ricketts TC, 3rd: **The comparative retention of National Health Service Corps and other rural physicians. Results of a 9-year follow-up study.** *JAMA* 1992, **268:**1552-1558.

17. Pathman DE, Konrad TR, Ricketts TC, 3rd: **Medical education and the retention of rural physicians.** *Health Serv Res* 1994, **29:**39-58.

18. Pathman DE, Konrad TR, Ricketts TC, 3rd: **The National Health Service Corps experience for rural physicians in the late 1980s.** *JAMA* 1994, **272:**1341-1348.

19. Pathman DE, Konrad TR: **Minority physicians serving in rural National Health Service Corps sites.** *Med Care* 1996, **34:**439-454.

20. Rosenblatt RA, Saunders G, Shreffler J, Pirani MJ, Larson EH, Hart LG: **Beyond retention: National Health Service Corps participation and subsequent practice locations of a cohort of rural family physicians.** *J Am Board Fam Pract* 1996, **9:**23-30.

21. Cullen TJ, Hart LG, Whitcomb ME, Rosenblatt RA: **The National Health Service Corps: rural physician service and retention.** *J Am Board Fam Pract* 1997, **10:**272-279.

22. Xu G, Veloski JJ, Hojat M, Politzer RM, Rabinowitz HK, Rattner S: **Factors influencing physicians' choices to practice in inner-city or rural areas.** *Acad Med* 1997, **72:**1026.

23. Xu G, Fields SK, Laine C, Veloski JJ, Barzansky B, Martini CJ: **The relationship between the race/ethnicity of generalist physicians and their care for underserved populations.** *Am J Public Health* 1997, **87:**817-822.

24. Singer JD, Davidson SM, Graham S, Davidson HS: **Physician retention in community and migrant health centers: who stays and for how long?** *Med Care* 1998, **36:**1198-1213.

25. Rabinowitz HK, Diamond JJ, Veloski JJ, Gayle JA: **The impact of multiple predictors on generalist physicians' care of underserved populations.** *Am J Public Health* 2000, **90:**1225-1228.

26. Rabinowitz HK, Diamond JJ, Markham FW, Paynter NP: **Critical factors for designing programs to increase the supply and retention of rural primary care physicians.** *JAMA* 2001, **286:**1041-1048.

27. Mofidi M, Konrad TR, Porterfield DS, Niska R, Wells B: **Provision of care to the underserved populations by National Health Service Corps alumni dentists.** *J Public Health Dent* 2002, **62:**102-108.

28. Brooks RG, Mardon R, Clawson A: **The rural physician workforce in Florida: a survey of US- and foreign-born primary care physicians.** *J Rural Health* 2003, **19:**484-491.

29. Porterfield DS, Konrad TR, Porter CQ, Leysieffer K, Martinez RM, Niska R, Wells B, Potter F: **Caring for the underserved: current practice of alumni of the National Health Service Corps.** *J Health Care Poor Underserved* 2003, **14:**256-271.

30. Probst JC, Samuels ME, Shaw TV, Hart GL, Daly C: **The National Health Service Corps and Medicaid inpatient care: experience in a southern state.** *South Med J* 2003, **96:**775-783.

31. Holmes GM: **Does the National Health Service Corps improve physician supply in underserved locations?** *Eastern Economic Journal* 2004, **30:**563-581.

32. Pathman DE, Fryer GE, Green LA, Phillips RL: **Changes in age-adjusted mortality rates and disparities for rural physician shortage areas staffed by the National Health Service Corps: 1984-1998.** *J Rural Health* 2005, **21:**214-220.

33. Holmes GM: **Increasing physician supply in medically underserved areas.** *Labour Economics* 2005, **12:**697-725.

34. Pathman DE, Fryer GE, Jr., Phillips RL, Smucny J, Miyoshi T, Green LA: **National Health Service Corps staffing and the growth of the local rural non-NHSC primary care physician workforce.** *J Rural Health* 2006, **22:**285-293.

35. Rittenhouse DR, Fryer GE, Jr., Phillips RL, Jr., Miyoshi T, Nielsen C, Goodman DC, Grumbach K: **Impact of Title VII training programs on community health center staffing and National Health Service Corps participation.** *Ann Fam Med* 2008, **6:**397-405.

36. Weiss LD, Wiese WH, Goodman AB: **Scholarship support for Indian students in the health sciences: an alternative method to address shortages in the underserved area.** *Public Health Rep* 1980, **95:**243-246.

37. Holmes JE, Miller DA: **A study of 138 return service scholarship applications awarded by the Oklahoma Physician Manpower Training Commission.** *J Okla State Med Assoc* 1985, **78:**384-388.

38. Lapolla M, Brandt EN, Jr., Barker A, Ryan L: **State public policy: the impacts of Oklahoma's physician incentive programs.** *J Okla State Med Assoc* 2004, **97:**190-194.

39. Pathman DE, Konrad TR, King TS, Spaulding C, Taylor DH: **Medical training debt and service commitments: the rural consequences.** *J Rural Health* 2000, **16:**264-272.

40. Dunbabin JS, McEwin K, Cameron I: **Postgraduate medical placements in rural areas: their impact on the rural medical workforce.** *Rural Remote Health* 2006, **6:**481.

41. Jackson J, Shannon CK, Pathman DE, Mason E, Nemitz JW: **A comparative assessment of West Virginia's financial incentive programs for rural physicians.** *J Rural Health* 2003, **19 Suppl:**329-339.

42. Pathman DE, Konrad TR, King TS, Taylor DH, Jr., Koch GG: **Outcomes of states' scholarship, loan repayment, and related programs for physicians.** *Med Care* 2004, **42:**560-568.

43. Ross AJ: **Success of a scholarship scheme for rural students.** *S Afr Med J* 2007, **97:**1087-1090.
